# Protein–Protein Interactions and Structure of Heat-Set Gels Based on Pea Protein and Egg White Mixtures

**DOI:** 10.3390/gels11030176

**Published:** 2025-02-27

**Authors:** Jian Kuang, Pascaline Hamon, Jeehyun Lee, Said Bouhallab, Eliane Cases, Remi Saurel, Valérie Lechevalier

**Affiliations:** 1 Institut National pour la Recherche Agronomique et Environnement (INRAE), L’Institut Agro Rennes-Angers, UMR STLO, 35042 Rennes, France; jian_kuang95@yeah.net (J.K.); pascaline.hamon@institut-agro.fr (P.H.); jeehyun.lee@institut-agro.fr (J.L.); said.bouhallab@inrae.fr (S.B.); 2 Université Bourgogne Franche-Comté, Institut Agro, INRAE, UMR PAM 1517, 21000 Dijon, France; eliane.cases@institut-agro.fr (E.C.); remi.saurel@institut-agro.fr (R.S.)

**Keywords:** gelation properties, egg white protein, pea protein isolate, protein interactions

## Abstract

The substitution of animal proteins with plant-based ones to fit environmental and economic demands is a challenge in gel applications. This study examined the thermal elation of mixtures of pea protein isolate (PPI) and egg white proteins (EWPs) at different PPI/EWP weight ratios (100/0, 75/25, 50/50, 25/75, 0/100) at pH 7.5 and 9.0. Viscoelastic and texture properties of the composite gels, along with the microstructure and molecular interactions involved in the gel network, were investigated. Except for PPI-EWP 100/0 at pH 9.0, all systems gelled with increasing gel hardness, springiness, and cohesiveness when EWP content increased. This was explained by the microstructure of the gels, wherein the presence of PPI enhanced the formation of aggregates embedded in the EWP network, thus loosening it. The rheological properties of the mixed gels were primarily influenced by the EWP network, involving disulfide bonds. However, upon the addition of PPI, hydrogen bonds and hydrophobic interactions predominated and the structure of the gel became more sensitive to pH as electrostatic repulsions interfered. Adjusting the ratio of PPI/EWP allows the production of gels with varying textures, and suggests the possibility of partially substituting egg white with pea proteins in food gel formulation.

## 1. Introduction

To meet the increasing demand for protein [1] and environmental sustainability, there is a necessity to boost protein transition by expanding the range of plant-based protein products. However, substituting animal proteins with plant-based ones in food formulations is not straightforward, as it requires knowledge of both bio-functional (digestibility, allergenicity, etc.) and techno-functional (foaming, emulsifying, gelling) properties of these ingredients. While plant proteins present environmental and health benefits, their techno-functional properties are often inferior to those of animal proteins.

Consequently, the use of combinations of plant and animal proteins has become increasingly appealing in the formulation of food products due to economic advantages, as well as interesting nutritional, functional, and organoleptic properties [2,3]. Gelling properties, in particular, are among the crucial functional properties of proteins, providing unique textures, sensations, and flavors in food products [4,5].

As an alternative to animal proteins, pulse proteins such as yellow pea (*Pisum sativum* L.) proteins are gaining attention due to their low price and allergen- and gluten-free composition [6,7,8]. Peas are one of the most widely cultivated legumes globally (4th after soybean, peanuts, and dry beans) and are consumed worldwide [9]. As a byproduct of the starch industry, pea proteins are a low-cost, readily available protein source. Pea seeds contain four main protein fractions: globulins (55–65% of total proteins), albumins (18–25%), prolamins (4–5%), and glutelins (3–4%) [10]. Thanks to this protein diversity, the amino acid content of pea proteins is higher than that of the proteins of reference recommended by the FAO for adults [11]. This makes pea proteins a good alternative to animal proteins from a bio-functional point of view.

During the heating process, proteins undergo unfolding and aggregation until self-supporting networks are formed. Multiple types of molecular interactions, such as hydrogen bonding and dipole–dipole, hydrophobic, and electrostatic interactions, are involved during the thermal aggregation and gelation of pea globulins [12,13]. The contribution of disulfide bonds in these heat-induced phenomena seems limited [13,14,15,16,17]. Consequently, pea proteins show moderate gelling properties with the minimum concentration to obtain a firm gel ranging from 14% to 20% depending on the extraction method used [18]. Combining pea proteins with more efficient gelling proteins could thus improve their use as gelling ingredients.

Egg white proteins have been extensively used in the food industry due to their ability to form gels with favorable nutritional and texture properties [19,20,21]. While their gelling properties in single-protein systems are well-documented [22], there is less available data on the gelation mechanisms of egg white protein when mixed with other protein sources. Su et al. [23] and Zhang et al. [5] studied the gelling properties of mixtures of soybean proteins with egg white and egg yolk/whole egg, respectively. While the mixture of egg white with soybean showed an increase in gel springiness and water-holding capacity, phase separation also occurred, leading the authors to favor whole egg or egg yolk over egg white. More recently, Alavi et al. [24] studied the acid gelation of mixed soluble aggregates of egg white protein and hemp seed protein isolate. While no gels were formed from the thermal aggregates of hemp seed protein isolate alone, the 25/75 egg white protein/hempseed protein ratio formed self-supporting gels.

Co-gelation of pumpkin seed proteins with egg white proteins was carried out by Tomczynska-Mleko et al. [25] to improve the nutritional value of the plant-based gel thanks to the essential amino acids present in egg white. The supplementation of pumpkin seed proteins with egg white proteins (80/20 *w*/*w*) resulted in more homogenous gels with a stronger microstructure and better water-binding capacity.

Since cross-linking between proteins could enhance the gel properties, Wu et al. [26] combined soy protein isolate or pea protein isolate with egg white to maximize gel texture, particularly its resistance to freezing. The addition of plant proteins (2.5% *w*/*w*) improved gel hardness, chewiness, gumminess, and water-holding capacity after freeze–thawing. The network structure of the gels with plant proteins was more orderly and regular after freezing. This was attributed to the increased contribution of disulfide bonds, hydrophobic interactions, and hydrogen bonds to the gel network in the presence of plant proteins.

In a previous study, Kuang [27] demonstrated that the heat-induced behavior of the egg white protein (EWP)/pea protein isolate (PPI) mixture was governed by egg white proteins, regardless of the egg white content in the mixture. However, the presence of pea proteins delayed the self-association of egg white proteins, especially at pH 9.0, the natural pH of egg white, suggesting the possibility of obtaining gels with a wider range of textures.

Thus, the present study aimed to investigate the comparative gelling, texture, and microstructure properties of composite gels based on mixed PPI-EWPs at various weight ratios from pure egg white protein gels to pure pea protein gels at both pH 7.5 and 9.0. Those pH values were chosen as they correspond to the pH of freshly laid egg white and 8 to 10 days stored egg white, respectively The objective was to highlight conditions (PPI-EWP ratio, pH) that could enable the substitution of some of the egg white protein content by pea proteins without breaking down the gel properties. Additionally, the intermolecular interactions involved in the heat-induced composite gels were also examined to explain the macroscopic behavior of the gels.

## 2. Results and Discussion

### 2.1. Thermal Gelation and Viscoelastic Properties of PPI-EWP Gels

**Figure 1:**
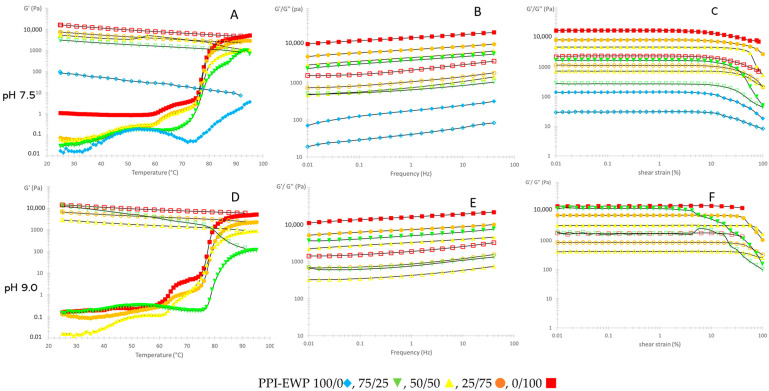
(**A**,**D**) The storage modulus of PPI-EWP gels during heating from 25 to 95 °C (full symbols), then cooling to 25 °C at 2 °C/min (empty symbols) (1 Hz, 0.1% strain) at pH 7.0 and 9.5; (**B**,**E**) Changes in storage (full symbols) and loss modulus (empty symbols) with frequency after cooling PPI EWP gels (25 °C, 0.1% strain) at pH 7.0 and 9.5; (**C**,**F**) changes in storage and loss modulus with increasing shear strain (25 °C, 1 Hz) at pH 7.0 and 9.5.

Temperature sweeps were performed using small amplitude rheology to understand the sol–gel transition behavior of the different protein suspensions upon thermal treatment. Typical storage modulus (G′) vs. temperature curves for 100% PPI, 100% EWP, and PPI-EWP mixtures at pH 7.5 and 9.0 are shown in Figure 1A,D. The final (after cooling) G′ and tan(δ) (loss factor) values of the respective protein gels are reported in Table 1.

Before heating, all samples exhibited a relatively low G′ that increased during heating due to the thermal denaturation and aggregation of the proteins, leading to the formation of a solid viscoelastic 3D protein network after cooling (tan(δ) < 1), except for the 100% PPI mixture at pH 9.0, which did not form a self-supporting gel under those conditions (Figure 1A). At this alkaline pH, far from the isoelectric point of pea proteins (pI ~ 4.5–4.8), strong electrostatic repulsions between negatively charged proteins prevented the formation of a cohesive protein network, resulting in only a viscous suspension for the 100% PPI sample [28]. At pH 7.5, the 100% PPI sample gelled, but the final G′ was lower than 100 Pa, significantly lower than the values obtained for the EWP-containing gels (>3000 Pa) (Table 1). Regardless of pH, mixtures containing more than 50% EWPs exhibited a two-step increase in G′ around 60 and 85 °C, previously attributed to the denaturation/aggregation of ovotransferrin (OVT) and ovalbumin (OVA), respectively [28]. When EWPs represented less than 50% of the mixture, a one-step increase was observed between 80 and 90 °C, consistent with the denaturation temperature of PPI globulins [28].

**Figure 2:**
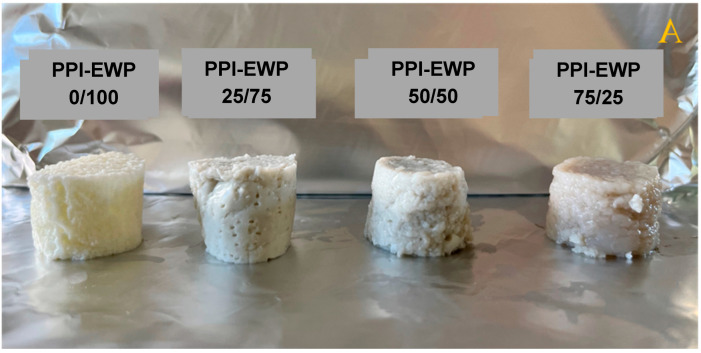

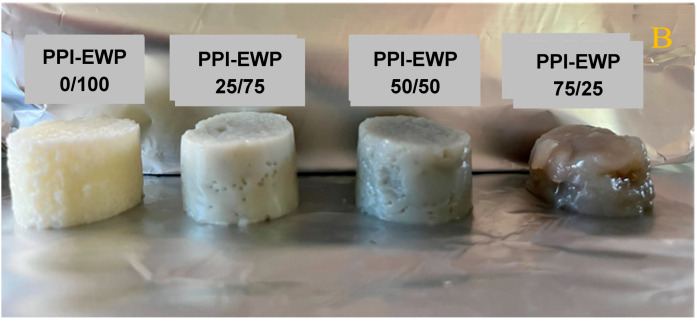
Photographs of PPI-EWP gels at the different weight ratios at pH 7.5 (**A**) and pH 9.0 (**B**).

The final G′ value of the gelled systems significantly increased with an increasing EWP content, except for the 75/25 PPI-EWP mixture at pH 9 (Table 1). Surprisingly, this last sample presented a final G′ value comparable to the 25/75 PPI-EWP sample at the same pH. However, a very high standard error was observed for this sample. Its G′ value was low at the end of heating and it increased primarily during cooling (about 90-fold) compared to other samples (about 3–4-fold). These observations indicated very unstable behavior for this mixture, having a final heterogeneous gel structure that could not be specified as a self-supporting gel network (Figure 2B). Moreover, as will be shown later, this sample contains aggregates over 10 µm in size that can cluster in the gap of the rheometer explaining the high standard deviation on the measurement.

While the G′ values of the gels containing at least 50% EWPs seemed independent of pH, tan(δ) was significantly lower at pH 9.0 compared to pH 7.5, indicating a more viscous contribution at lower pH (Table 1). Tan(δ) value also decreased with increased EWP content and reached 0.13 and 0.11 for the 100% EWP sample at pH 7.5 and 9.0, respectively. These values were characteristic of weak gels (tan(δ) > 0.1) [29], and weaker gels were formed with the gradual addition of PPI to the mixture as tan(δ) increased. The “weak” character of the gels was confirmed by the frequency sweep data presented in Figure 1B. For all samples, G′ and G″ were frequency-dependent, and increased with increasing frequency, confirming the formation of weak viscoelastic gels (G′ > G″). Physical gels are typically uncrosslinked and are characterized by entanglements and weak chemical associations within the macromolecular network with a time-scale dependence upon mechanical [30]. Similar behavior has been reported for soy– [23] and oat–egg white [31] protein mixtures.

Additionally, strain sweeps were performed on the final gels, and typical curves are presented in Figure 1C. All curves exhibited distinct linear and non-linear viscoelastic regions. In the linear viscoelastic region (LVR), the gels deformed elastically, with the storage modulus (G′) higher than the loss modulus (G″), indicating the gel-like nature of the samples. Beyond that region, G′ decreased due to the breakdown of the network structure. The corresponding yield points (YP) were determined and are reported in Table 2.

With the increased proportion of PPI in the mixture, the YP first increased up to 50% PPI (the 50/50 weight ratio sample presented a maximum at both pHs) and then decreased. This behavior could be explained by the variable structure of the gels at the microscopic level depending on the percentage of each protein type in the mixture and will be discussed in Section 2.3. In general, lower YP indicates weaker connections in the protein network, leading to earlier network rupture upon oscillatory deformation.

The linear response region also increased with pH values, suggesting that the protein gel network had more structural strength and was more elastically deformable at pH 9.0, in agreement with the previous works of Handa et al. [32] and Alleoni and Antunes [33]. Both groups observed that the hardness and elasticity of the EWP gels were higher at pH 9.0 than at pH 7.0. These authors attributed this behavior to the increased proportion of S-OVA in egg white during storage at pH 9.0, suggesting that S-OVA could improve the hardness of albumen gels. Additionally, more recently, Somaratne et al. [34] found that the hardness of egg white gels at pH 9.0 was higher than that at pH 5.0, due to a more homogeneous network at pH 9.0 compared to the heterogeneous protein network composed of larger aggregate particles at pH 5.0.

### 2.2. Macrostructure of PPI-EWP Gels

The macrostructure of the gels was characterized by analyzing their appearance and performing a texture profile analysis (TPA). The appearance of PPI-EWP gels at the different weight ratios is shown in Figure 2. Since PPI alone barely gelled (at pH 7.5) or did not gel at all (at pH 9.0), the 100% PPI samples are not presented.

The color of the gels obtained from the different PPI-EWP mixtures changed with the increasing proportion of PPI, ranging from pale yellow to light brown and dark brown, at pH 7.5 (Figure 2A) and pH 9.0 (Figure 2B), respectively. These color changes may be due to the presence of phenolic compounds in PPI samples as suggested by Zhou et al. [35] for RuBisCo gels. The color of PPI-EWP gels at pH 9.0 was darker than those at pH 7.5, in agreement with the observations of Zhang et al. [36] for gellan gum gels in the presence of tea polyphenols.

**Figure 3:**
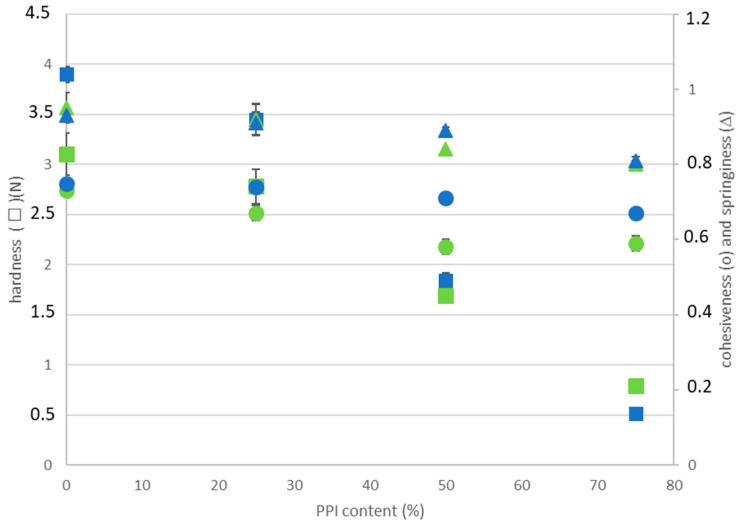
Hardness (squares), cohesiveness (circles), and springiness (triangles) of PPI-EWP gels as a function of PPI content at pH 7.5 (green) and 9.0 (blue).

The texture of the gels was evaluated through TPA. Hardness and springiness were typically regarded as relevant measures of gel performance [19,24]. The changes in TPA parameters (hardness, springiness, and cohesiveness) of the gels are presented in Figure 3.

At both pH levels, 100% EWP gels exhibited the highest gel hardness, which significantly decreased with the increasing proportion of PPI content (from 0 to 75%) in the initial mixture. This is consistent with the previous viscoelastic data where G′ decreased and tan(δ) increased with increasing PPI content. A similar trend has been observed for egg white–hemp seed protein mixtures [24] and egg white–soy protein composite gels at higher protein concentrations [23]. From 50% EWP content in the system upward, the hardness was higher at pH 9.0 compared to pH 7.5. This result was also consistent with lower tan(δ) and higher YP values, respectively, at pH 9.0, as observed in the previous section.

Similar effects related to gel composition were observed for springiness and cohesiveness, both of which slightly decreased with higher PPI content. These parameters represent textural qualities associated with gel elasticity and the ability to maintain an intact network structure [32,37].

In summary, the presence of PPI modified the texture of the gels, decreasing their hardness and increasing their brittleness, as suggested by the decrease in both springiness and cohesiveness. Such results have already been described for other plant protein gels [35], where aggregates and/or protein–protein interactions of varying nature and strength were observed.

### 2.3. Microstructure of PPI-EWP Gels

**Figure 4:**
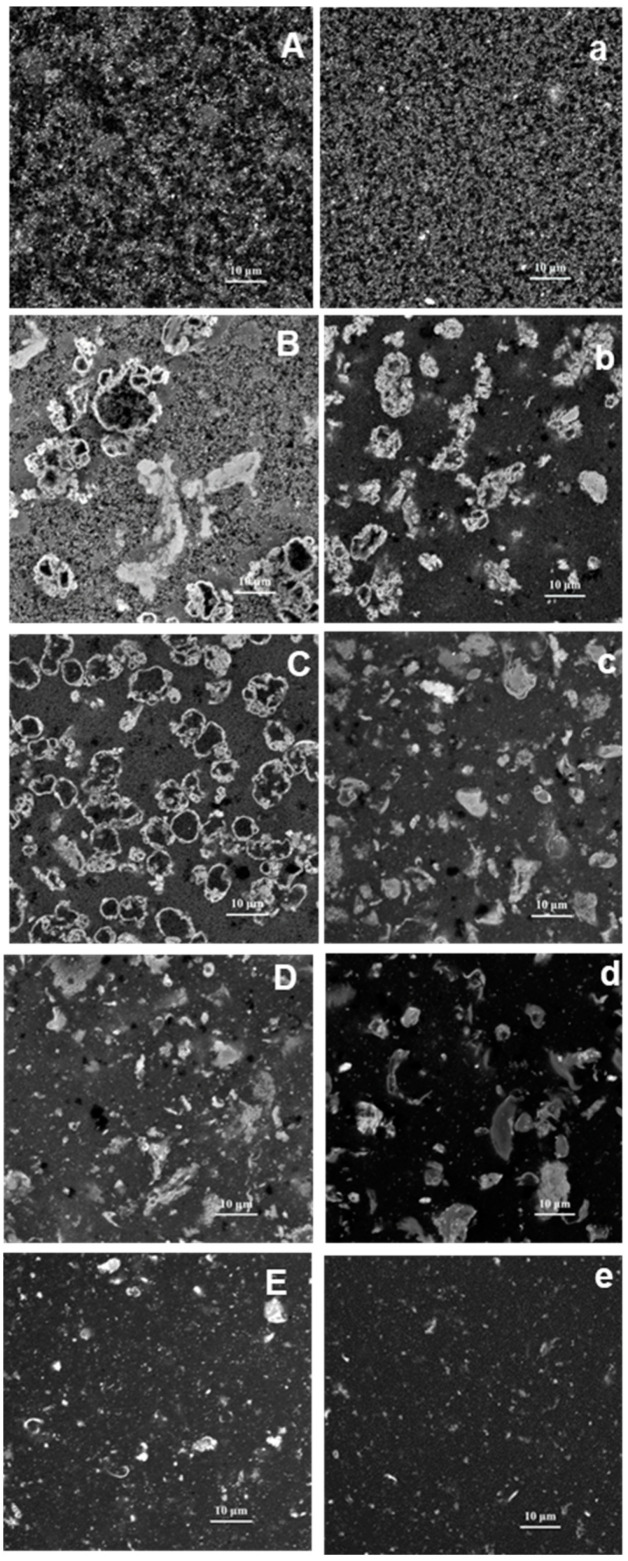
CLSM images visualizing the microstructure of PPI-EWP gels (0/100 (**A**,**a**), 25/75 (**B**,**b**), 50/50 (**C**,**c**), 75/25 (**D**,**d**); 100/0 (**E**,**e**)) at pH 7.5 (**left**) and pH 9 (**right**) (magnification ×63).

The microstructure of PPI-EWP gels was observed using confocal microscopy. Figure 4 shows the microscopic observations of 10% (*w*/*w*) mixed protein gels at various PPI-EWP weight ratios (0/100, 25/75, 50/50, 75/25, 100/0) at pH 7.5 and 9.0. Proteins are visible in gray and white on confocal micrographs, while pores containing the aqueous phase appear in black. It is worth noting that both EWP and PPI were labeled, preventing their differentiation in these images.

For the pure EWP system (Figure 4A,a), the microstructural organization of the gel composed of fine aggregates, differed noticeably between pH 7.5 and 9.0. At pH 9.0, the EWP gel showed a denser and more homogeneous protein network compared to pH 7.5, where the protein network was more porous and loosely packed. This result is consistent with previously published SEM and cryo-TEM data, which showed a granular (pH 7) vs. smooth (pH 9) EWP gel microstructure [38,39], and CLSM observations, which demonstrated a more homogeneous structure of EWP gels at pH 9 than at pH 5 [40]. The differences in gel structures at both pH levels may be attributed to the varying behavior of OVA and OVT during gelation at pH 7.5 and 9.0 [38]. At pH 7.5, OVT was near its isoelectric point (pI) (6.5), promoting the formation of random and spherical aggregates, while OVA, far from its pI (4.5), began to form linear branched aggregates [38]. As a result, in this case, the egg white gel was made up of a variety of aggregated structures, namely, OVT spherical aggregates dispersed in the protein network of OVA linear branched aggregates. Van der Plancken et al. [41] emphasized that at pH 9, the net protein charge and electrostatic repulsions were significantly enhanced, and the activation energy required for protein unfolding was reduced. In this case, proteins tended to unfold to form a homogeneous protein network rather than spherical aggregates [39].

Figure 4E,e show the microstructure of heated PPI at pH 7.5 and 9.0, respectively. At pH 9.0, protein particles and small aggregates were poorly interconnected, indicating no gel formation, as mentioned previously, whereas at pH 7.5, a denser protein network with gel-like properties was observed. Additionally, larger particles were seen at pH 7.5 (Figure 4E), while only spaced small particles were visible at pH 9.0 (Figure 4e). The higher repulsive force between protein particles at high pH may explain the formation of smaller aggregates with insufficient interconnections to form a solid network.

Different structures were observed for the three mixed protein systems at both pHs (Figure 4B–D,b–d). For the 25/75 PPI-EWP gels at pH 7.5 (Figure 4B), large irregular-shaped aggregates having heterogeneous size (from ten to several hundred µm) formed, surrounded by a white homogeneous protein network. It was assumed that this homogeneous part of the network was formed by egg proteins, as it composed most of 50% of the mixture and the structure looked like the 100% egg white protein gels.

With 50% of PPI in the mixtures (Figure 4C) at the same pH, more numerous spherical aggregates (~10 µm) with black holes were present, and the surrounding network area decreased. With a higher concentration of PPI in PPI-EWP mixtures, the gel structure became more heterogeneous, forming random protein clusters of smaller size and irregular shape (Figure 4D). In contrast, PPI-EWP gels formed at pH 9.0 (Figure 4b–d) exhibited some differences. When EWPs were the dominant component (Figure 4b), the corresponding gel showed some large aggregates (~10–20 µm) resembling brain-like structures, surrounded by a continuous network similar to pure EWP gel. When PPI comprised 50% of the mixture (Figure 4c), the gel contained more numerous protein clusters of smaller size (<10 µm). When PPI was the dominant component (Figure 4d), very irregular clusters were dispersed within a poorly defined continuous phase. Similar observations regarding mixed gels were previously reported by Kornet et al. [42], who found that whey protein-PPI gels contained large clusters at high pea protein content. Silva et al. [43] demonstrated that mixtures of micellar caseins and PPI at pH 5.8 formed gels with protein clusters, whereas more homogeneous gels were obtained for individual proteins. McCann et al. [3] and Roesch and Corredig [44] observed a discontinuous network in soy protein-whey protein gels at a total protein concentration of around 6%, indicating phase separation, while Gómez-Mascaraque and Pinho [45] found a microgel structure between soy and whey protein gels.

As evidenced from the structural observations of the mixed gels mentioned above, the network structure of these gels was not as dense and regular as that of egg white, with the formation of large clustered aggregates that did not exist in the pure PPI systems. In the mixtures, it was assumed that the EWPs could form the basic architecture of the protein network, and gelation was accompanied by the formation of protein aggregates, which could be either pure PPI aggregates or mixed aggregates consisting of pea globulins and some EWPs. Notably, positively charged lysozyme (LYS) can form complexes with pea proteins [46]. The total or partial phase separation between EWPs and PPI could result from depletion or thermodynamic incompatibility effects [47,48,49]. Although thermodynamic incompatibility is commonly described between food proteins and polysaccharides, these phase separation phenomena could occur between proteins of different natures [50]. In our systems, these phenomena would likely be amplified by the lower gelation temperature of OVT. Indeed, our group has previously shown that an initial gel point appeared at a temperature <59 °C in egg white-based systems, with this early gelation attributed to OVT [28]. The primary gel network formed would tend to exclude other protein aggregate particles that form later during heating, consisting primarily of nascent pea protein aggregates that reassemble into large clusters. The structural differences observed at pH 9.0 would be due to the repulsive forces between protein particles at this pH limiting pea proteins’ self-association. In this case, smaller aggregates would form with less ability to interconnect. These results aligned with the decrease in G′ and TPA parameters observed as the proportion of PPI increased in the mixtures as described in the previous sections. The aggregates observed via CLSM could weaken the primary EWP network, explaining the changes in gel texture. It is assumed that phase separation phenomena between proteins could increase interconnections within the dominant EWP network, while more protein clusters affected the network’s continuity and weakened the gel. This counterbalanced phenomenon could explain the maximum observed for YP in strain weep experiments presented in Section 2.1. Micro-phase separation first extended the elastic deformability region for low proportions of PPI in the mixtures, while a lower EWP concentration in the continuous network at higher PPI proportions negatively affected the gel’s elastic strength.

### 2.4. Intermolecular Interactions Involved in PPI-EWP Gels

Typical protein gels can be stabilized by both non-covalent and covalent forces. For instance, Chang and Chen [51] demonstrated that hydrophobic interactions, disulfide bonds, and hydrogen bonds stabilize EWP thermal gels. To assess the types of interactions involved in PPI-EWP mixture-based gels at pH 7.5 and 9.0, a dissociation approach was employed and compared with predicted effects. The use of urea, propylene glycol, DTT, and guanidinium-HCl as dissociating agents allowed the evaluation of interactions between proteins in various gels. Table 3 summarizes the reported effects of urea, DTT, propylene glycol, and guanidinium–HCl on hydrogen bonds, disulfide bonds, and hydrophobic interactions, respectively. In this approach, it is assumed that the more the gel was dissolved in the presence of a chemical agent, the more the agent was able to affect the corresponding interactions and release soluble protein particles.

#### 2.4.1. Effect of Dissociating Agents on 100% PPI and 100% EWP Gels

**Figure 5:**
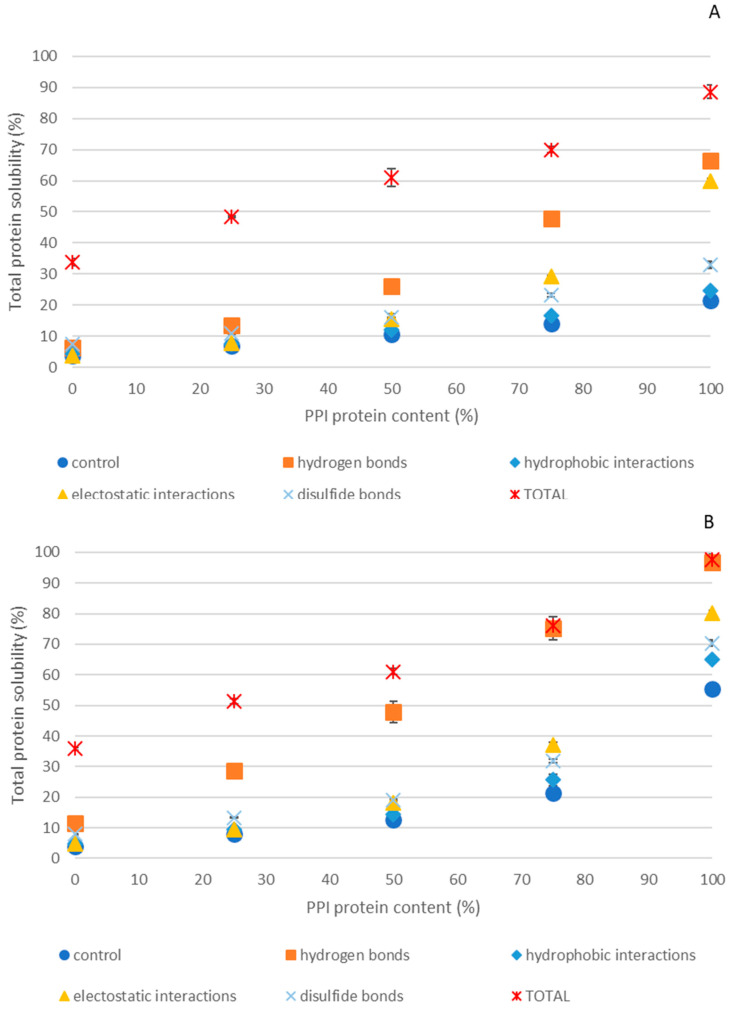
Protein solubilization from PPI-EWP gels as a function of PPI protein content at pH 7.5 (**A**) and 9.0 (**B**). Control (blue points) was obtained by solubilization in 100 mM Tris-HCl, hydrogen bonds (orange squares) were obtained by solubilization in 100 mM Tris-HCl + 8 mM urea, hydrophobic interactions (blue diamond) were obtained by solubilization in 100 mM Tris-HCl + 20% PG, electrostatic interactions (yellow triangles) were obtained by solubilization in 100 mM Tris-HCl + 2 M Gu-HCl; disulfide bonds (blue crosses) were obtained by solubilization in 100 mM Tris-HCl + 100 mM DTT and Total (red crosses) were obtained by solubilization in 100 mM Tris-HCl + 8 mM urea + 20% PG + 2 M Gu-HCl + 100 mM DTT.

Figure 5 shows the percentage of proteins solubilized by dissociating agents for both PPI-EWP gels at both pH 7.5 (Figure 5A) and 9.0 (Figure 5B).

Dissolution of the gels in 100 mM Tris buffer (used as a control) allows us to understand which fraction of the protein system is dissociated in the absence of any dissociating agent. It can be hypothesized that this solubility corresponds to protein particles not bound to the gel network or that certain interactions were weakened by the buffer, releasing some part of the protein material. Tris (C_4_H_11_NO_3_) is a very polar molecule with one amine and three hydroxyl groups (a weak base) and a pKa of 8.3, close to the two pH values studied. At a concentration of 100 mM, the properties of the molecule could affect hydrogen and ionic bonds, which would explain the partial protein dissociation from the gels in this buffer. The 100% EWP gel was poorly dissociated in this buffer (approximately 4% at both pH values), while the solubility of the 100% PPI gel increased to approximately 21% at pH 7.5 and 55% at pH 9.0. This suggests that, while most of the EWP was strongly retained in the gel network, PPI was more easily released into the solution, especially at pH 9.0 where its high electronic charge may favor disruption of hydrogen and ionic bonds by the Tris buffer.

Compared to the control, no significant differences were observed for hydrophobic interactions regardless of the sample or pH, and it was therefore not commented further.

Regardless of the dissociating agent used (including the control) and the pH, the amount of protein dissociated from the 100% PPI gel was consistently much higher than from the 100% EWP gel (Figure 5), suggesting there are fewer or weaker interactions in PPI gels than in EWP gels. At pH 9.0, the quantity of protein dissociated from both the 100% PPI gel (more accurately described as a coagulum in this case) and the 100% EWP gel was generally higher than at pH 7.5 (Figure 5A vs. Figure 5B). This suggests the presence of a greater amount of (i) low-energy interactions and/or (ii) proteins not associated with the protein network at pH 9.0. In comparison, the protein solubility of 100% EWP gels at both pH levels remained low for all dissociating agents (≤11.5%), suggesting that even though some interactions were affected by the chemical agents, the gel particles released remained insufficiently soluble, indicating the presence of strong interactions.

In 100% PPI gels, urea, guanidinium–HCl, and DTT significantly increased the quantity of solubilized protein regardless of the pH (Figure 5) with DTT having the smallest effect. This suggests that hydrophobic interactions, hydrogen bonds, and, to a lesser extent disulfide bonds were involved in PPI gels. These results align with those of Sun and Arntfield [13], who reported that hydrophobic interactions and hydrogen bonds mainly contributed to heat-induced pea protein gelation with 0.3 M NaCl at pH 5.65, while disulfide bonds played a minor role in gel formation. Tanger et al. [60] also confirmed that the main protein interactions in pea protein gels were non-covalent regardless of pH and ionic strength.

In contrast, in 100% EWP gels, only urea and DTT had a significant effect on total protein solubilization for both pHs (Figure 5), suggesting the predominance of hydrophobic and disulfide bonds in these gels. This result is consistent with previous works by Huang et al. [61] and Wang et al. [62], who found that disulfide bonds in egg white gel outnumbered hydrophobic effects. Jin et al. [63] also reported that disulfide bonds play the primary role in heat-induced EWP gel formation, followed by hydrophobic interactions, hydrogen bonds, and ionic bonds, regardless of heating duration.

The simultaneous application of all four dissociating agents led to a dramatic increase in protein solubilization for all samples, indicating the synergistic effect of the dissociating agents, regardless of pH and gel type. However, the EWP gels remained particularly insoluble, with only around 35% of proteins solubilized regardless of the pH. Finally, adding all the chemical agents simultaneously did not lead to complete solubilization of the gelled systems except in the case of PPI at pH 9.0, with a total solubility reaching 97.5% (noting that no self-supporting gel was formed under these conditions).

#### 2.4.2. Effect of Dissociating Agents on PPI-EWP Mixed Gels

The protein solubility of PPI-EWP mixed gels at different weight ratios and pH values (7.5 and 9.0) increased in the presence of dissociating agents as the proportion of PPI protein in the mixed gels increased (Figure 5) The mixed gels exhibited an intermediate behavior between the 100% EWP and 100% PPI systems in terms of chemical dissociation. In all cases, the gels at pH 9.0 were more easily dissociated than those at pH 7.5, as the higher pH promoted greater repulsive forces within the protein network during gel formation due to the increased protein charge at a more alkaline pH (Figure 5A vs. Figure 5B). Compared to rheological and texture data, the trend toward higher solubilization in the presence of chemical agents among the gelled systems corresponded to the formation of weaker gels.

EWP-based gels were weakly dissociated up to the 50/50 ratio, even when the chemical agents were used simultaneously, confirming that EWPs played a dominant role in the structure of the gels, consistent with the CLSM observations (Figure 4). These gels were especially sensitive to urea and DTT regardless of the pH, indicating the significant role of hydrogen bonds, hydrophobic interactions, and disulfide bonds in the structure of these mixed gels. In contrast, when the mixed gels were rich in PPI (PPI-EWP 75/25), urea proved to be the most effective dissociating agent (~48/75% solubilization), followed by guanidinium–HCl (~29/37% solubilization) at pH 7.5/9.0 (Figure 5) with a minor contribution from disulfide bonds (~23/32% solubilization). This result suggests a combination of non-specific and lower-energy interactions, similar to what was observed in 100% PPI gels, with hydrogen bonds and hydrophobic interactions being dominant.

## 3. Conclusions

- **proposal of a mechanism for gelation of PPI-EWP mixtures**

Combining the results of texture analysis, dynamic rheology, microscopy, and chemical dissociation data, we propose the following mechanism regarding the heat-induced gelation of PPI-EWP mixtures at pH 7.5 and 9.0. We hypothesize that the heat-set gels obtained from the PPI-EWP mixtures consist of a primary network of egg white proteins embedding large aggregates of pea proteins or mixed PPI-EWP, induced by a phase separation phenomenon. This is suggested by gel microstructure observations that show a continuous protein network, very similar to that of the pure EWP system, with irregular protein clusters of varying sizes embedded within it. This hypothesis is further supported by the viscoelastic data indicating an initial gelation point around 55 °C when heating the protein mixtures containing at least 50% EWP, attributed to the denaturation of OVT at a lower temperature than the other proteins. At higher temperatures (>60 °C), the denaturation of other proteins leads to the formation of large protein aggregates, likely driven by thermodynamic incompatibility, depletion, and/or steric exclusion phenomena. These aggregates likely involve pea globulins as they are not observed in pure EWP systems, although the contribution of other egg white proteins (OVA, LYS, etc.) to these aggregates cannot be excluded. The smaller size of the dispersed protein particles at pH 9 compared to pH 7.5 could be explained by increased repulsive forces between proteins at the more alkaline pH, which limit self-association. Additionally, viscoelastic data and texture parameters show that stronger, more rigid, and cohesive gels are formed as the proportion of EWPs increases in the initial protein suspensions. The existence of a continuous protein network, primarily composed of egg white proteins, enhances the tightness of the gels dominated by strong hydrophobic interactions and disulfide bonds. Furthermore, the 25/75 PPI-EWP mixture produced gels with a texture similar to that of pure EWP gels, especially at pH 9.0, suggesting that a partial substitution of EWPs by PPI could be viable in gel applications.

This study provides a deeper understanding of the gelation properties of hybrid protein systems and will contribute to improving the design of composite protein ingredients or new plant-based food products.

## 4. Materials and Methods

### 4.1. Samples Preparation

Fresh eggs were sourced from a local market in Dijon (France), stored at 4 °C, and utilized 15 days prior to the expiration date. The fresh liquid egg white was meticulously separated from the egg yolk and chalaza. The resulting egg white was then transferred to a beaker and gently homogenized using a magnetic stirrer for 2 h at room temperature. The total protein content of the egg white was determined using the Kjeldahl method (N = 6.25), yielding a value of 10.2% *w*/*w* on a dry basis.

Pea globulins were extracted from smooth yellow pea flour (*P. sativum* L.) supplied by Cosucra (Warcoing, Belgium), following the method described by Kuang et al. [46]. The resulting protein powder, designated as PPI, contained 89% *w*/*w* proteins on a dry basis. Prior to use, the PPI was solubilized in distilled water to achieve a 10% protein content. The dispersion was then agitated at 350–400 rpm for 3 h at 4 °C to ensure complete hydration of the proteins. The pH of the protein suspensions was subsequently adjusted to pH 7.5 or pH 9.0 using 0.1 M HCl or NaOH before each test, without altering the dispersion’s concentration. The insoluble protein fraction was considered negligible.

All other reagents and chemicals, procured from Sigma-Aldrich (St-Quentin Fallavier, France), were of analytical grade.

### 4.2. Gel Preparation

Protein suspensions (10%, *w*/*w*) were prepared at pH 7.5 and 9.0 (adjusted with 0.1 M HCl or NaOH) from initial egg white proteins (EWPs) and stock suspensions of pea protein isolate (PPI) to obtain 100% PPI, 100% EWPs, and PPI-EWP mixtures at three weight ratios (75/25, 50/50, and 25/75). The protein suspensions were transferred into glass vials and heated from 25 to 95 °C (at a rate of 5 °C/min) in a water bath, then maintained at 95 °C for 15 min. Subsequently, the vials were cooled to room temperature in an ice bath and stored at 4 °C overnight to ensure complete gelation.

### 4.3. Small-Strain Dynamic Rheology

Each protein suspension of PPI, EWPs, and their mixtures at pH 7.5 or 9.0 was loaded into a rheometer MCR 302e (Anton Paar, Graz, Austria) equipped with a 50 mm parallel plate geometry. Approximately 1 mL of each sample was transferred to the lower plate of the parallel plate geometry of the rheometer. The upper plate was lowered to achieve a gap width of 1.0 mm. A thin layer of light mineral oil was added to the well of the upper plate geometry, and a solvent trap cover was used to prevent evaporation during heating, thus maintaining a water-saturated atmosphere at the surface of the sample. The following heating protocol was applied: the sample was initially equilibrated at 25 °C for 3 min, then heated at a rate of 2 °C/min and cooled at a rate of 5 °C/min over a temperature range of 25–95–25 °C under a shear strain of 1% and a frequency of 1 Hz. Subsequently, a frequency sweep over a range of 0.01–40 Hz at 1% strain and a strain sweep from 0.01 to 100% strain at 1 Hz were performed at 25 °C. The storage modulus (G′) and loss modulus (G″) were measured during temperature, strain, and frequency sweeps. The loss factor or tangent delta (tan δ = G″/G′) was also calculated (calculated at 1 Hz, 1% strain), as well as the linear viscoelastic region (LVR). The LVR was calculated as described in Figure 1 for the PPI-EWP mixture at a weight ratio of 25/75. The intersection of the two lines on both sides of the inflection point was the maximum strain without causing permanent deformation, called the yield point. Rheological data were collected for every degree change during heating and cooling. Samples were run in triplicates

### 4.4. Texture Profile Analysis

For texture profile analysis (TPA), all samples were prepared as described in Section 2.2 in plastic tubes with a diameter of 40 mm (Krehalon, Deventer, The Netherlands). Forty grams of sample suspensions were heated from 25 to 95 °C in a water bath and maintained at this temperature for 15 min, then cooled down with ice to room temperature and stored at 4 °C overnight. Cylindrical gels with a diameter of 40 mm were sliced using a die cutter at a height of 20 mm and placed on the platform of a TA-XT Plus (Lloyd Instruments, Ametek company, Berwyn, IL, USA) equipped with a 5 N load cell and a cylindrical probe with a diameter of 12 mm (SMS-P/35). TPA tests were conducted at a test speed of 0.5 mm/s, and a deformation in compression of 37.5% was applied. A time of 10 s was allowed to elapse between the two compression cycles. All samples were prepared in duplicate and tested twice.

The hardness, springiness, and cohesiveness of PPI-EWP gels were determined according to the method described by Bourne [64]. Hardness was defined as the maximum peak force during the first compression cycle. Springiness was defined as the degree of recovery of gels after decompression to their initial shape, measured by the distance of the detected height during the second compression divided by the original compression distance. Cohesiveness was calculated as the area of work during the second compression divided by the area of work during the first compression. Data were analyzed using Texture Expert software version 1.22 (Stable Micro Systems).

### 4.5. Confocal Microscopy (CLSM)

Sample preparation and microscopy analysis were conducted following the procedures outlined by Somaratne et al. [40] and Kuang et al. [28] with minor adjustments. Four hundred microliters of each 10% protein solution at pH 7.5 or 9.0 were dispensed into 1 mL Eppendorf tubes. They were then mixed with 12 μL of 1% (*w*/*v*) Fast Green solution. Subsequently, the entire sample solution was gently loaded into the well of a chamber slide (Ibidiμ-Slide 8 well Uncoated system, Ibidi, Grafelfing, Germany). The system was covered with the provided lid and securely wrapped with Parafilm around the lid gap. Additionally, aluminum foil was used to prevent the photo-bleaching of fluorescent molecules. Finally, the systems were placed into the IBIDI system and subjected to heating as described for gel preparation in Section 4.2.

The mixture gels labeled with Fast Green were visualized using a Zeiss LSM 880 Inverted confocal microscope (Carl Zeiss AG, Oberkochen, Germany) equipped with the Airyscan detection unit. The prepared slide was positioned on a ×63 oil-immersion objective (NA = 1.4) in the thermo-regulated chamber of the microscope, set at 20 °C. A He/Ne laser with a wavelength of 633 nm was employed, and images were captured using the Airyscan detector in super-resolution mode with the zoom set at 1.8. Zen Black 2.1 (version 13.0.0.0) software was used to process the acquired datasets using the 2D mode with the default settings of the Airyscan processing function.

### 4.6. Gel Dissolution by Dissociating Agents

Four different extracting reagents were used to analyze protein–protein interactions contributing to gelation. Samples were prepared according to the methods proposed by Liu and Hsieh [65] and Chen et al. [66] with some modifications. A 100 mM Tris buffer solution (Tris) at pH 7.5 or pH 9.0 was used as a control (i). Tris buffer containing 8 M urea (ii), 2 M guanidinium hydrochloride (GuHCl) (iii), or 20% propylene glycol (PG) (iv) was used to extract proteins by affecting non-covalent interactions. Tris buffer containing 100 mM dithiothreitol (DTT) was used to extract proteins by reducing disulfide bonds (v). Tris buffer containing 6 M urea, 100 mM DTT, 2 M guanidinium hydrochloride (GuHCl), and 20% propylene glycol (PG) was used to extract proteins by dissociating all disulfide and non-covalent bonds as a second control (vi).

Following the protocol outlined by Chen et al. [65], gel samples (~2.5 g), prepared as described in Section 2.2, were incubated in individual extractants (~40 mL), stirred for 1 h at 25 °C, and homogenized for 1 min at 10,000 rpm using a homogenizer (Ultra Turrax^®^ IKA T25 Digital, IKA, Staufen, Germany). The samples were then centrifuged (16,000 rpm, 30 min, 4 °C). The supernatants were collected, filtered using a 0.45 μm filter, weighed, and diluted with the same extractant for protein assay. The protein content in the dilutions was measured using a commercial Coomassie Plus (Bradford) protein assay kit (λ = 660 nm) obtained from Sigma-Aldrich (St-Quentin Fallavier, France), with BSA as the standard. The solubilized protein content was then calculated as follows (Equations (1) and (2)):

(1) $$Total\;protein\;solubility\;\left(\%\right)=\frac{protein\;content\;in\;gel\;supernatant\;solution}{protein\;content\;in\;gels}\times 100$$

(2) $$Net\;protein\;solubility\;in\;dissociating\;buffer\;\left(\%\right)=total\;protein\;solubility-protein\;solubility\;in\;Tris$$

At least three extractions were conducted and analyzed for each sample.

### 4.7. Statistical Analysis

Differences between samples were studied by analysis of variance (one-way ANOVA). Significance was set at *p* < 0.05. Tukey’s post hoc least significant differences method was used to describe means with 95% confidence intervals. The statistical analyses were performed using Statistica software, version 12 (Tulsa, OK, USA).

## Figures and Tables

**Table 1:** Final G′ and tan (δ) of PPI-EWP gels at the different weight ratios and pH after temperature sweep (0.1% strain, 1 Hz frequency).

PPI-EWP Ratio	G′ (Pa)		Tan (δ)	
	pH 7.5	pH 9.0	pH 7.5	pH 9.0
0/100	15,115 ± 632 ^aA^	14,446 ± 413 ^aA^	0.135 ± 0.002 ^aA^	0.115 ± 0.001 ^aB^
25/75	7284 ± 192 ^bA^	7204 ± 281 ^bA^	0.138 ± 0.001 ^aA^	0.118 ± 0.003 ^aB^
50/50	4725 ± 324 ^cA^	4182 ± 440 ^cA^	0.151 ± 0.002 ^bA^	0.134 ± 0.004 ^bB^
75/25	3446 ± 331 ^cA^	9237 ± 3249 ^bB^	0.157 ± 0.003 ^bA^	0.158 ± 0.008 ^cA^
100/0	97 ± 4 ^d^	no gel	0.227 ± 0.005 ^c^	no gel

All data ae given as mean ± SD (n ≥ 3). Means in a column bearing the same lowercase letter are not significantly different (*p* < 0.05). Means in a row with the same uppercase letter are not significantly different (*p* < 0.05).

**Table 2:** Yield point (%) of PPI-EWP gels at the different weight ratios at pH 7.5 and 9.0.

PPI-EWP Ratio	Yield Point (%)	
	pH 7.5	pH 9.0
0/100	5.5 ± 0.1 ^a^	16.6 ± 0.6 ^a^
25/75	9.7 ± 0.6 ^b^	41.6 ± 5.4 ^b^
50/50	11.4 ± 0.6 ^b^	52.3 ± 2.0 ^b^
75/25	3.9 ± 0.2 ^a^	9.7 ± 3.8 ^a^
100/0	5.6 ± 0.3 ^a^	no gel

All data are given as mean ± SD of triplicate measurements. Means in a column bearing the same letter are not significantly different (*p* < 0.05).

**Table 3:** Effect of various reagents on molecular forces existing in protein structures.

	Non-Covalent Bonds			Covalent Bonds	References
	Ionic Effect/Electrostatic Interactions	Hydrophobic Interactions	Hydrogen Bonds	Disulfide Bonds	
Dithiothreitol (DTT)				Disrupt	[13,52,53]
Guanidinium–HCl (GuHCl)	Disrupt	Weaken	Disrupt		[13,53,54]
Propylene glycol (PG)	Promote	Disrupt	Promote		[55,56,57]
Urea		Disrupt	Disrupt		[56,58,59]

## Data Availability

The raw data supporting the conclusions of this article will be made available by the authors upon request.
